# Arabidopsis calcium-dependent protein kinase 3 regulates actin cytoskeleton organization and immunity

**DOI:** 10.1038/s41467-020-20007-4

**Published:** 2020-12-04

**Authors:** Yi-Ju Lu, Pai Li, Masaki Shimono, Alex Corrion, Takumi Higaki, Sheng Yang He, Brad Day

**Affiliations:** 1 Department of Plant, Soil and Microbial Sciences, Michigan State University, East Lansing, MI 48824 USA; 2 Department of Plant Biology, Michigan State University, East Lansing, MI 48824 USA; 3 International Research Organization for Advanced Science and Technology (IROAST), Kumamoto University, Kumamoto, 860-8555 Japan; 4 Department of Energy Plant Research Laboratory, East Lansing, MI 48824 USA; 5 Howard Hughes Medical Institute, Michigan State University, East Lansing, MI 48824 USA

**Keywords:** Plant cell biology, Plant immunity, Effectors in plant pathology

## Abstract

Pattern-triggered immunity and effector-triggered immunity are two primary forms of innate immunity in land plants. The molecular components and connecting nodes of pattern-triggered immunity and effector-triggered immunity are not fully understood. Here, we report that the Arabidopsis calcium-dependent protein kinase CPK3 is a key regulator of both pattern-triggered immunity and effector-triggered immunity. In vitro and in vivo phosphorylation assays, coupled with genetic and cell biology-based analyses, show that actin-depolymerization factor 4 (ADF4) is a physiological substrate of CPK3, and that phosphorylation of ADF4 by CPK3 governs actin cytoskeletal organization associated with pattern-triggered immunity. CPK3 regulates stomatal closure induced by flg22 and is required for resistance to *Pst* DC3000. Our data further demonstrates that CPK3 is required for resistance to *Pst* DC3000 carrying the effector AvrPphB. These results suggest that CPK3 is a missing link between cytoskeleton organization, pattern-triggered immunity and effector-triggered immunity.

## Abstract (web summary)

Remodelling of the actin cytoskeleton occurs during plant immune responses to pathogens. Here Lu et al. show that this process requires the calcium-dependent kinase CPK3 which phosphorylates actin depolymerizing factor 4 and is required for both PAMP and effector-triggered immunity in Arabidopsis.

## Introduction

The actin-depolymerizing factor (ADF) and cofilin family of proteins function as key regulators of actin cytoskeletal dynamics^1,2^, responsible for controlling numerous aspects of cytoskeletal organization, including filament growth, stability, organization, and disassembly. Additionally, ADF activity influences the size and distribution of the actin monomer pool, a process that controls the rapid assembly, growth, and orientation of the cytoskeletal network within a cell. Underpinning each of these processes is phosphorylation, a key step controlling ADF-actin association and depolymerization activity^3^. In mammalian systems, the cofilin phospho-switch is regulated by LIM (LIMK1 and LIMK2) and TES (TESK1 and TESK2) protein kinases^4,5^. In plants, this process is proposed to be functionally similar; however, despite the identification of several ADF-phosphorylating kinases^6–8^, the kinase responsible for ADF phosphorylation relevant to plant-pathogen interactions remains enigmatic.

Recent studies demonstrate a role for the plant actin cytoskeleton in pathogen recognition and the activation of both PTI and ETI^9–17^. As a function of ETI, ADF4 has been shown to be required for immune signaling mediated by the nucleotide-binding leucine-rich repeat resistance (R) protein RPS5, a process that is initiated following recognition of the type-III effector (T3E) AvrPphB, a cysteine protease^13,16^. A key step in the activation of RPS5 is AvrPphB cleavage of the receptor-like cytoplasmic kinase PBS1 (AvrPphB Susceptible-1) by AvrPphB^18,19^, a mechanism hypothesized to be associated with R-protein destabilization and immune activation. In addition to R-protein activation, recent work has identified ~10 PBS1-like kinases that are cleaved by AvrPphB^20^, among which includes the immune kinase BIK1 (*Botrytis*-induced kinase-1)^21^. This is significant, as it implicates BIK1 and additional immune kinases as potential regulators of distinct pathogen recognition events in plants, integrating immune responses activated through multiple pattern recognition receptors, including FLS2 (Arabidopsis flagellin receptor^22^), EFR (receptor for bacterial Ef-Tu^23^), and CERK1 (chitin receptor^23^). As a broader function of this signaling cascade, recent work has shown that the actin cytoskeleton is not only required for the activity of the plant immune system, but that actin is actively targeted by pathogens during infection^15,24^. In the case of PTI, it has been demonstrated that pathogen-associated molecular pattern (PAMP) recognition requires the function of the plant actin cytoskeleton, presumably to initiate downstream signaling associated with resistance^10^. However, the mechanistic connection between the requirement of ADF4 in RPS5-mediated ETI, AvrPphB-mediated cleavage of immune kinases, and PTI remains a puzzle.

In the current work, we present the identification of a kinase responsible for ADF4 phosphorylation and subsequent control of actin filament association and depolymerization during immune signaling. Using a combination of in vitro and in vivo kinase assays coupled with a mass spectroscopy-based approach, we show that the Arabidopsis calcium-dependent protein kinase, CPK3, is responsible for ADF4 phosphorylation, a process that regulates actin cytoskeletal dynamics. We also demonstrate that CPK3 is required for proper stomatal actin filament organization and PTI-induced guard cell gating. In addition, analysis of the immune signaling function of the *cpk3-2* mutant revealed that inoculation of plants with *Pst* DC3000 expressing AvrPphB (*Pst*-AvrPphB) phenocopies the *adf4* mutant^13,16^, with significant increases in the *in planta* growth of the pathogen and an absence of the hypersensitive response (HR). In total, these data support a model whereby CPK3 plays an important role in PTI and, through its phosphorylation of ADF4 is also required for ETI activation by pathogen effectors (e.g., cysteine protease AvrPphB) that destabilize immune receptor-guarded proteins.

## Results

### CPK3 phosphorylates the actin-depolymerizing factor ADF4

**Fig. 1:**
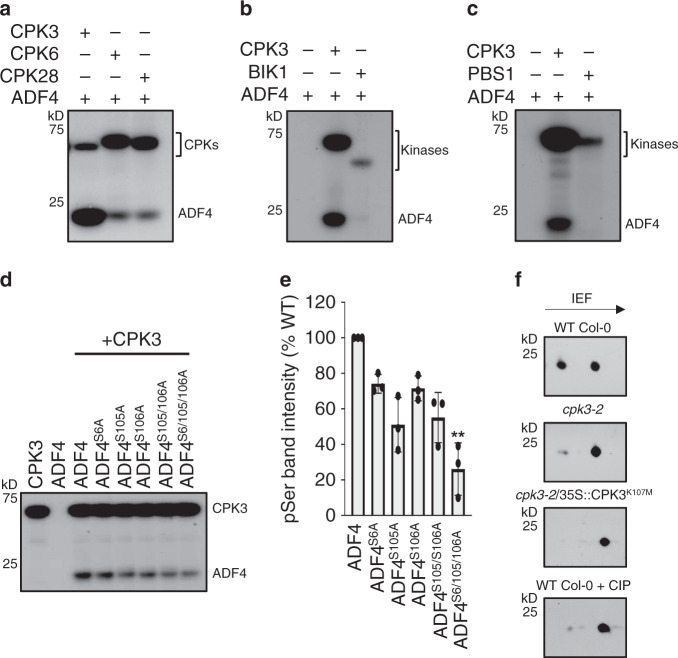
CPK3 phosphorylates the Arabidopsis actin-depolymerizing factor ADF4. In vitro kinase assays using recombinant CPK3, CPK6, CPK28, and ADF4. **a** CPK3 predominantly phosphorylates ADF4 as compared to CPK6 and CPK28. The receptor-like cytoplasmic Ser/Thr kinases **b** BIK1 and **c** PBS1 do not phosphorylate ADF4. **d** CPK3 predominantly phosphorylates ADF4 at amino acid residues Ser^105^. **a**–**d** Experiments were repeated three times. **e** Mean ± SD of densitometry scans from three independent biological replicates of the kinase assays shown in **d**. Data is represented as a percentage of WT ADF4 phosphorylation. Asterisks denote standard deviation of the mean compared to WT ADF4 (one-way ANOVA, *p-value < 0.1; **p-value < 0.01). **f** Two-dimensional gel electrophoresis analysis of HA-tagged ADF4 expressed in WT Col-0, *cpk3-2*, the kinase inactive-expressing line *cpk3-2*/CPK3^K107M^ and CIP-treated WT Col-0 protoplasts. Arrow indicates the direction of isoelectric focusing (IEF). Experiments were repeated two times.

Previous work identified a genetic interaction between ADF4-dependent changes in actin cytoskeletal organization and the activation of the AvrPphB-RPS5 immune signaling node^13,16^. To define the mechanism(s) underpinning changes in actin cytoskeletal organization during pathogen infection, and moreover, the regulation of ADF4, we sought to identify the kinase responsible for ADF4 regulation. Leveraging previously published data showing phosphorylation of ADF1 by CPK3^7^, we first performed in vitro kinase assays using recombinant CPK3, CPK6, and CPK28—each of which represent a distinct clade of the CPK family—to interrogate the role of CPK in the regulation of ADF4. Recombinant ADF4 was preferentially phosphorylated by CPK3, while ADF4 was nominally phosphorylated by CPK6 and CPK28 (Fig. 1a, Supplementary Fig. 1, and Supplementary Fig. 2a). As expected, the kinase-inactive variant, CPK3^K107M^, did not autophosphorylate (Supplementary Fig. 2b), nor did it phosphorylate ADF4 (Supplementary Fig. 2c).

To identify CPK3 phosphorylation sites on ADF4, we first employed an in silico-based approach, the result of which revealed high-confidence predictions for phosphorylation of ADF4 at Ser^105^, with moderate phosphorylation at Ser^106^, as well as minor phosphorylation events at Ser^6^ (Supplementary Fig. 3a). Mass spectroscopy analysis of in vitro kinase assays confirmed these predictions (Supplementary Fig. 3b, c). As a broader function of the interaction specificity between CPK3 and ADF4, we also tested the in vitro activity of the PTI-associated RLCK BIK1 (Fig. 1b and Supplementary Fig. 1b), as well as the ETI-associated immune RLCK PBS1 (Fig. 1c and Supplementary Fig. 1c) and found that neither kinase phosphorylated ADF4; this result was also confirmed by mass spectroscopy (Supplementary Fig. 4).

To validate the ADF4 phosphorylation residues, we used PCR mutagenesis to convert the predicted high-confidence phospho-serine residues (i.e., Ser^6^, Ser^105^, and Ser^106^) to alanine (i.e., S6A, S105A, and S106A), and again performed in vitro kinase assays. Using this approach, we were successful in identifying preferential phosphorylation of ADF4 by CPK3 at Ser^105^ (Fig. 1d, e and Supplementary Fig. 1d). To validate that phosphorylation of ADF4 by CPK3 occurs in vivo, we performed two-dimensional electrophoresis analysis of Arabidopsis protoplasts prepared from WT Col-0 and the *cpk3-2* mutant and transiently expressed the kinase substrate ADF4 containing an HA-epitope tag (i.e., ADF4-HA). As shown in Fig. 1f, ADF4-HA expressed in WT Col-0 protoplasts showed a robust phosphorylation. Conversely, and in support of our hypothesis, in protoplasts derived from the *cpk3-2* mutant, the kinase-dead CPK3 complementation line (i.e., *cpk3-2*/*35S::CPK3*^*K107M*^), and protoplasts treated with calf intestinal alkaline phosphatase (CIP), ADF4 phosphorylation was significantly reduced. Thus, both in vitro and in vivo phosphorylation assays confirm that ADF4 is a physiological substrate of CPK3.

**Fig. 2:**
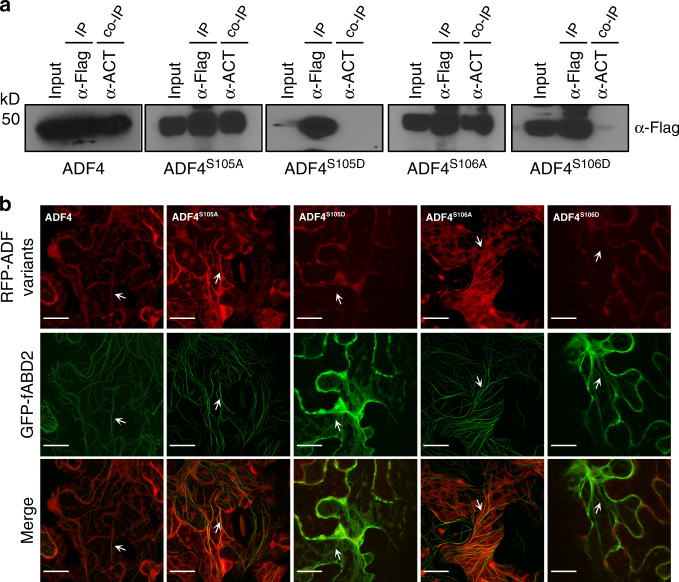
Ser-105 and Ser-106 are essential for ADF4 interaction with actin. **a** Co-immunoprecipitation of ADF4 phosphorylation variants with *Arabidopsis* actin following transient expression in *N. benthamiana*; the phosphomimetic variants do not co-immunoprecipitated with Actin. **b** In vivo fluorescence microscopy localization of ADF4 variants and actin-binding domain. Constructs were co-expressed in *N. benthamiana* as N-terminal mRFP fusion proteins for ADF4 variants and a C-terminal GFP fusion protein for fABD2 using *A. tumefaciens*-mediated transient expression. Arrows indicate co-localization of the fluorescence signal with F-actin. Scale bar = 10 μm. Experiments were performed three times with similar results, and 30 images were collected in total for each co-expressed construct.

Next, we asked if Ser^105^ and Ser^106^ are required for ADF4 association with actin in planta. To test this, we performed co-immunoprecipitation assays using WT ADF4, as well as the phospho-null (Ser to Ala) and phosphomimic (Ser to Asp) derivatives, with *Arabidopsis* actin-7 (ACT7) following transient expression in *N. benthamiana*. While ADF4 and the phosphor-null derivatives ADF4^S105A^, and ADF4^S106A^ interacted with ACT7, the phosphomimic variants ADF4^S105D^, and ADF4^S106D^ did not associate with actin (Fig. 2a and Supplementary Fig. 5). Similar results were observed by transiently expressing WT ADF4 and its phosphomimetic derivatives as red fluorescent protein (RFP) fusions proteins in *N. benthamiana* in combination with green florescent protein (GFP)-tagged actin-binding domain (fABD2). As shown, the phosphomimic derivatives ADF4^S105D^-RFP and ADF4^S106D^-RFP did not co-localize with fABD2, while ADF4^S6D^-RFPhad attenuated association (Fig. 2b and Supplementary Fig. 5). These data support a key role for these three serine residues in F-actin binding by ADF4.

### CPK3 is required for proper actin filament organization in epidermal pavement cells and stomatal guard cells during pathogen infection

**Fig. 3:**
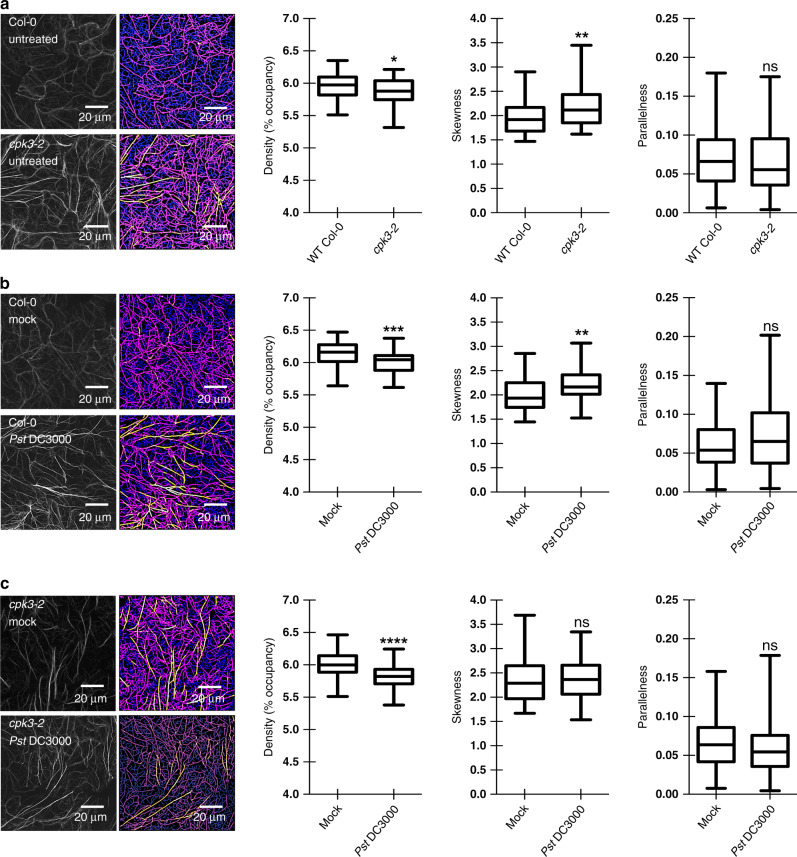
CPK3 activity modulates ETI- and PTI-induced changes in actin cytoskeletal organization. Quantitative analysis of actin cytoskeletal organization in epidermal pavement cells from **a** untreated WT Col-0 and the *cpk3-2* mutant, mock and *Pst* DC3000-inoculated **b** WT Col-0 and the *cpk3-2* mutant, and **c** flg22-elicited WT Col-0 and the *cpk3-2* mutant. Representative confocal images (left) and skeletonized images for quantitative evaluation (right) are shown. Images were collected from ≥75 cells. Experiments were performed three times from multiple, independent, biological replicates. The box and whiskers plots represent minimum and maximum values. The line in the box plot represents the median value and the boundaries demonstrate the 25th percentile (upper) and the 75th percentile (lower). The statistics is performed with two tailed Mann–Whitney *U*-test; *p-value < 0.05; **p-value < 0.01; ns, ***p < 0.001; ****p < 0.0001; ns, not significant.

Having identified CPK3 as an ADF4 kinase, we next investigated the effect of CPK3 on cytoskeletal organization. Specifically, we examined actin filament architecture in epidermal pavement cells of WT Col-0/*35S::GFP-fABD2* and *cpk3-2*/*35S*::*GFP-fABD2* plants. *cpk3-2* mutant plants showed significant decreases in filament occupancy (represented by density) and higher levels of bundling (represented by skewness) in cells from untreated plants. We did not detect a significant difference in the parallel organization of actin filaments (Fig. 3a). Following inoculation of seedlings with virulent *Pst* DC3000, WT Col-0/GFP-fABD2 plants showed a marked, statistically significant, increase in actin filament bundling (Fig. 3b), consistent with previous observations^15^. Conversely, following inoculation of *cpk3-2*/*35S*::*GFP-fABD2* with *Pst* DC3000, we did not observe an increase in actin filament bundling as compared to mock-inoculated *cpk3-2*/*35S::GFP-fABD2* (Fig. 3c). These data support a role for CPK3 in pathogen-induced actin filament (re)-organization. Interestingly, we identified a statistically significant increase in *CPK3* mRNA accumulation following flg22 treatment (Supplementary Fig. 6), indicating a genetic interaction between PTI signaling and CPK3.

Flg22-induced MAPK activation (Supplementary Fig. 7a), the generation of reactive oxygen species (ROS) (Supplementary Fig. 7b) and flg22-triggered priming (Supplementary Fig. 8) were not altered in the *cpk3-2* mutant compared to WT Col-0. Unexpectedly, however, we observed reductions in *FRK1, PR-1, WRKY70*, and *WRKY46* mRNAs in the *cpk3-2* mutant line following flg22 stimulation (Supplementary Fig. 9). At present, it is unknown if these reductions in mRNAs are due to alternations in actin filament dynamics, or are related to additional, direct or indirect functions of CPK3.

Previous work demonstrated that stomatal guard cell actin filament organization is altered in response to PTI signaling^10,14^. To determine if CPK3, through its phosphorylation of ADF4, exerts control over the steady-state cellular concentration of G- and F-actin, we assessed the relative abundance of actin in WT Col-0 and the *cpk3-2* mutant. As shown in Supplementary Fig. 10 the ratio of total G- and F-actin from 4-week-old plants was similar in both WT Col-0 and the *cpk3-2* mutant in both untreated and *Pst* DC3000-inoculated leaves. Conversely, upon elicitation of flg22-induced PTI, we observed a dramatic shift in the cellular ratios of G- and F-actin; however, we did not detect a significant difference in G- and F-actin ratios between WT Col-0 and the *cpk3-2* mutant. Thus, CPK3 does not appear to be required for maintenance of cellular G- and F-actin levels and/or the ratio of the two. Based on this, we surmise that CPK3′s primary role, as a function of the cytoskeleton, is the regulation of actin filament dynamics and organization via control of ADF4 activity.

**Fig. 4:**
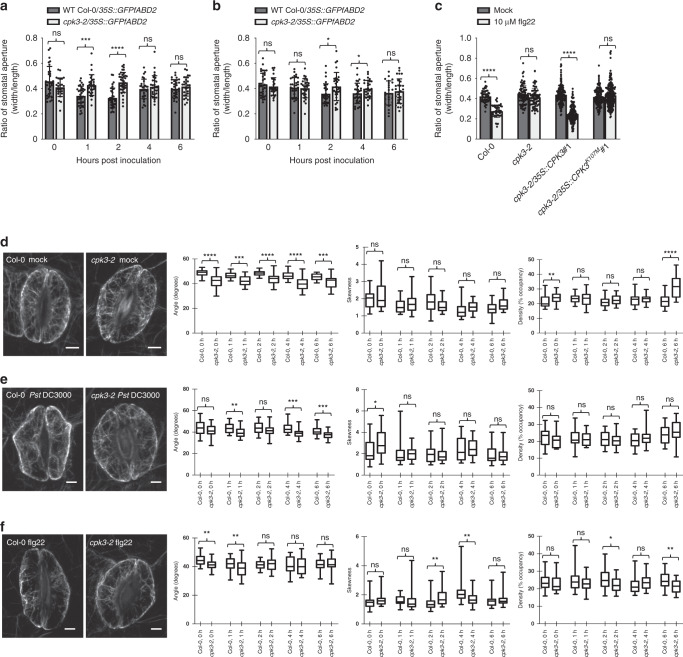
CPK3 activity is required for stomatal immunity and guard cell actin dynamics. Deletion of CPK3 compromises stomatal immunity. Guard cells from the *cpk3-2* mutant do not close in response to **a** flg22 or **b** *Pst* DC3000 treatment over a 6 h time course. Fourteen-day-old seedlings were stimulated with water or 10 μM purified flg22 peptide for 1 h, and images were collected by laser scanning confocal microscopy. **c** CPK3 kinase function is essential for guard cell response to flg22 treatment. Four-week-old WT Col-0, the *cpk3-2* mutant, the complementation lines with full kinase function (i.e., *cpk3-2/35S::CPK3*^*#1*^), and the kinase null line (*cpk3-2*/*35S::CPK3*^*K107M#1*^) were stimulated with 10 μM purified flg22 peptide for 1 h. Images were collected by laser scanning confocal microscopy, and stomatal apertures (width/length) were calculated using ImageJ. Error bars represent means ± SD from three technical replicates of three independent biological repeats (*n* ≥ 24). Statistical analysis was performed using a two tailed Mann–Whitney *U*-test; *p < 0.05; ***p < 0.001; ****p < 0.0001; ns, not significant). Guard cell actin parameters of WT Col-0/*35S::GFP-fABD2* and the *cpk3-2*/*35S::GFP-fABD2* mutant from 14-day-old seedlings inoculated with **d** mock, **e** 3 × 10^7^ CFU mL^−1^ *Pst* DC3000, and **f** 10 μM flg22. Left panels are representative images for guard cells. Stomatal images were collected over a 6 h time interval following inoculation. Images were collected from ≥24 cell pairs, and experiments were performed three times from multiple, independent, biological replicates. The box and whiskers plots represent minimum and maximum values. The line in the box plot represents the median value and the boundaries demonstrate the 25th percentile (upper) and the 75th percentile (lower). Statistical analysis was conducted using a two tailed Mann–Whitney U-test; *p-value < 0.05; **p-value < 0.01; ns, ***p < 0.001; ****p < 0.0001; ns, not significant.

To further define the role of CPK3 in actin filament organization and immune signaling, we undertook a comprehensive, and quantitative, evaluation of actin filament organization in guard cells from WT Col-0 and the *cpk3-2* mutant. As a baseline for PAMP and pathogen-induced reorganization of the actin filament array in stomatal guard cells, uninoculated, naïve WT Col-0 and *cpk3-2* mutant plants were first quantitatively evaluated for angular differences in filament orientation. This was achieved by measurements of mean angular difference between actin filament arrays and the stomatal pore edge. Interestingly, we observed that stomatal guard cells have a lower value in the *cpk3-2* mutant plant as compared to WT Col-0 (Fig. 4d), revealing that actin arrays in the *cpk3-2* mutant are predominantly longitudinal. In addition, skewness values, which represent the degree of filament bundling, were higher in the *cpk3-2* mutant than in WT Col-0 at 0 h (Fig. 4d–f). This result is consistent with our observation of filament organization in epidermal pavement cells (Fig. 3a). Additionally, following flg22 and *Pst* DC3000 inoculation, the angular difference in both WT Col-0 and the *cpk3-2* mutant showed a significant reduction in filament angles at 1 hpi (Fig. 4e, f), indicative of the initiation of stomatal closure^13^. After elicitation with 10 μM flg22 (Fig. 4f), incremental changes in skewness occurred at 2-to-4 hpi in WT Col-0, suggesting stomatal guard cell filament recovery, a process coincident with guard cell opening. In the *cpk3-2* mutant, however, changes in actin filament skewness and occupancy were minor.

Stomatal closure constitutes a functional output of PTI^25^. Based on previous work^13^, the altered actin filament dynamics and organization in the *cpk3-2* guard cell raised the possibility that the *cpk3-2* mutant are compromised in stomatal closure response during infection. To define the role of CPK3 in flg22 and *Pst* DC3000-induced stomatal closure, leaves from WT Col-0 and the *cpk3-2* mutant were elicited with flg22 and the stomatal guard cell response was evaluated. Following flg22 and *Pst* DC3000 elicitation, WT Col-0 stomata rapidly closed (Fig. 4a, b). However, elicitation of *cpk3-2* mutant plants with 10 μM flg22 did not induce stomatal closure (Fig. 4c); complementation of the *cpk3-2* mutant restored the flg22-induced closure response (Fig. 4c and Supplementary Fig. 11). Expression of the kinase-dead variant of CPK3 (i.e., *cpk3-2*/*35S::CPK3*^*K107M*^) did not restore flg22-induced stomata closure (Fig. 4c and Supplementary Fig. 11). These results demonstrate that activation of CPK3 is required for PTI-associated stomata closure in response to flg22 perception.

### CPK3 is required for both basal defense against Pst DC3000 and AvrPphB-triggered ETI

**Fig. 5:**
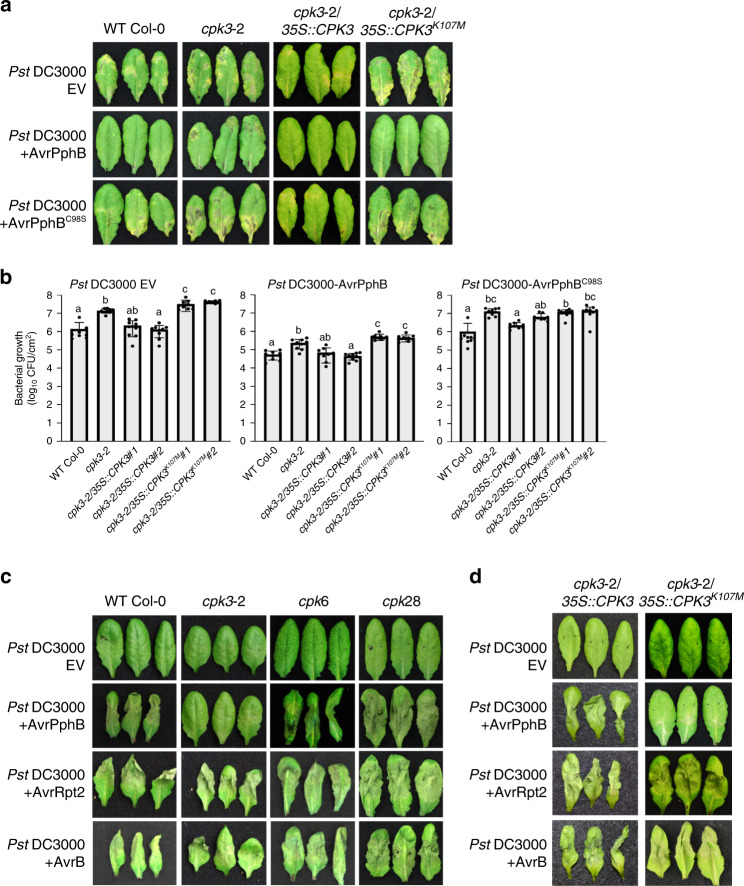
CPK3 is required for immunity to *Pst* DC3000-AvrPphB. a *CPK3* kinase function is required for disease resistance to *Pst* DC3000 and *Pst* DC3000-AvrPphB. Four-week-old plants were dip-inoculated with *Pst* DC3000 strains [3 × 10^7^ CFU mL^−1^]. Images were taken at 4 dpi. Three representative leaves are shown from *n* ≥ 30. All experiments were performed three times, with similar results. **b** In planta bacterial growth was enumerated at 4 days post-inoculation (dpi). Error bars represent means ± SD from three technical replicates of three independent biological repeats (*n* = 9, one-way ANOVA; *p* < 0.05; ns, not significant). **c** The *CPK3* mutant, *cpk3-2*, is specifically compromised in the activation of the *Pst* DC3000-AvrPphB-induced hypersensitive response (HR). Leaves from 4-week-old plants were infiltrated with *Pst* DC3000-AvrPphB [10^7^ colony-forming units (CFU mL^−1^)] using a 1-mL needleless syringe. Images were taken at 24 h post-inoculation (hpi). *CPK6* and *CPK28* mutants show WT levels of HR induction following *Pst* DC3000-AvrPphB inoculation. **d** *CPK3* kinase function is required for HR to *Pst* DC3000-AvrPphB. Four-week-old plants were infiltrated with *Pst* DC3000 strains [10^7^ CFU mL^−1^] using a 1-mL needleless syringe.

Having demonstrated a critical role of CPK3 as a regulator of ADF4-mediated actin filament dynamics and organization during PTI and stomatal closure, we next pursued experiments to gain insight into whether CPK3 contributes to the host resistance against pathogen in terms of or basal defense and ETI. To do this, we first inoculated WT Col-0, *cpk3-2*, the complementation lines *cpk3-2*/CPK3 and *cpk3-2*/CPK3^K107M^ with *Pst* DC3000. As shown, the *cpk3-2* mutant and *cpk3-2*/CPK3^K107M^ showed enhanced disease symptom development and significantly higher pathogen growth compared to WT Col-0 (Fig. 5a, b and Supplementary Figs. 12 and 13). This, together with additional data (e.g., Supplementary Fig. 7 and Supplementary Fig. 9), indicates that CPK3, including its kinase activity, is required for the activation of robustness basal defense signaling. We posit that this further illustrates the role of CPK3 in PTI-triggered actin reorganization and stomatal immunity.

To evaluate the role of CPK3 in ETI, and as an extension of our previous work with ADF4^13,15^, we next measured the in planta growth of *Pst* DC3000-AvrPphB. As shown in Fig. 5, we observed a significant increase in susceptibility in *cpk3-2*/CPK3 and *cpk3-2*/CPK3^K107M^ plant lines (Fig. 5a, b and Supplementary Fig. 13). Next, we evaluated the induction of the ETI-induced HR by infiltrating plants (WT Col-0, *cpk3-2, cpk6*, and *cpk28*) with *Pst* DC3000, *Pst* DC3000-AvrPphB, *Pst* DC3000-AvrRpt2, and *Pst* DC3000-AvrB (Fig. 5c and Supplementary Fig. 12). Consistent with previous reports showing a blocked HR in the *adf4* mutant^13,16^, we also observed that the HR was abrogated in the *cpk3-2* mutant following *Pst* DC3000-AvrPphB inoculation, yet unaffected in *Pst* DC3000-AvrRpt2 and *Pst* DC3000-AvrB inoculated plants (Fig. 5c). Further evaluation of the specificity of CPK3 using the complementation lines revealed a restoration in disease symptom development in *cpk3-2*/CPK3, but not the kinase inactive-expressing line *cpk3-2*/CPK3^K107M^ (Fig. 5d). Taken together, these data demonstrate that CPK3 also plays a significant role in AvrPphB-triggered ETI. To investigate whether CPK3 is an upstream signaling component of ADF4-dependent AvrPphB-mediated resistance, we generated a *cpk3-2*/*adf4-2* double mutant and performed bacterial growth curve assays to evaluate ETI. As shown, these data reveal that both *cpk3-2* and *adf4-2* mutant lines are more susceptible that WT Col-0 against *Pst* DC3000-AvrPphB, and additionally, that there is not an additive effect (i.e., increased susceptibility) in the *cpk3-2*/*adf4-2* double mutant (Supplementary Fig. 13c). This data supports our hypothesis that CPK3 and ADF4 likely function within the same immune signaling pathway.

To further define the link between AvrPphB and the activation of resistance through the modulation of host actin cytoskeletal organization, we asked if the function(s) of AvrPphB, RPS5, and/or PBS1 are associated with an actin-containing complex, in vivo. We did not detect a specific interaction between either PBS1 or RPS5 with actin (Supplementary Fig. 14). However, and quite surprisingly, we identified an interaction between the catalytically inactive AvrPphB^C98S^ and plant actin. This finding suggests another possible link between the HR cell death-inducing activity of the AvrPphB-RPS5 signaling node and a concomitant disruption in actin filament architecture via CPK3-ADF4 (Supplementary Fig. 15a). However, we did not observe a direct impact on actin filament bundling or depolymerization using recombinant AvrPphB protein and purified non-muscle actin, in vitro (Supplementary Fig 15b, c). We posit that these data indicate that during RPS5-mediated ETI, CPK3 may not simply function in inhibiting the activity of ADF4 by phosphorylation, but likely involves additional signaling processes, which contribute to the overall depolymerization of actin filaments. In total, these results signify a functional link between AvrPphB, CPK3, and the requirement for actin remodeling during *Pst* DC3000-AvrPphB that would require further investigation.

## Discussion

Previous studies have implicated a role for the plant cytoskeleton during pathogen infection and the activation of immunity^10–17,24,26^. However, key gaps remain in defining a role for cytoskeletal organization during plant defense. In the current study, we undertook an in vivo genetic and cell biology-based approach to define the mechanisms underpinning of the control of actin cytoskeletal organization during pathogen infection. As presented, the data herein provide compelling evidence that ADF4 is a physiological substrate of CPK3, and that phosphorylation of ADF4 by CPK3 is likely a required step in the activation of immune signaling. As a link to the requirement for specific immune-associated changes in cytoskeletal organization, our data also demonstrate that CPK3 is required for pathogen-induced changes in the host actin cytoskeleton. Taken together, the data presented herein support a mechanism for CPK3-mediated control of actin-dependent immune regulation, a process that is targeted by Pst DC3000-AvrPphB during infection. Based on this, we propose that CPK3 represents a missing link between actin cytoskeletal organization, PTI, and ETI, and is a key virulence target during pathogen infection. In short, our data support a model whereby changes in actin cytoskeletal organization contribute to the activation of immunity during pathogen infection.

In Arabidopsis, ~70 actin-binding proteins (ABPs) are required for filament organization^9^. In humans, more than 200 ABPs are required^27–29^. Among these ABPs, members of the actin-depolymerizing factor and cofilin family are key regulators of actin cytoskeletal organization^25,26^. ADFs are present in all eukaryotes, with 11 functional ADFs in Arabidopsis, varying in tissue and developmental expression^30,31^. In humans, actin depolymerization is controlled via the activity of 2-cofilins and 1-ADF^28^, and in total, it has demonstrated that depolymerization activity is regulated by a variety of factors, including phosphorylation, the expression and spatial distribution of cofilin, the ratio of globular (G)- versus filamentous (F)-actin, and the relative expression levels and activity of other ABPs. In short, it is hypothesized that actin depolymerization is dependent upon the cellular equilibrium of actin, which includes the spatial and temporal expression of ABPs and their regulators^27^. As an example of the importance of cofilin/ADF in human disease models, disruptions in the function of this class of protein is associated with numerous diseases, including Alzheimer’s, Parkinson’s, and DNA damage repair in cancer^29,32^.

In contrast to humans, plants have an expanded number of ADF genes, which is hypothesized to impart functional specificity based on temporal, tissue, and developmental patterns of expression^31^. Of the 11 ADFs in Arabidopsis, several reports have demonstrated a role for ADF4 in immune signaling in response to bacterial and fungal pathogen infection^13,16,20^. In total, these studies utilized a suite of complementary in vitro biochemical and in vivo microscopic methods for the characterization of ADF4, demonstrating a bona fide, in vivo, actin filament depolymerization function^16,33^. This study, together with previous studies, advances our understanding of the role of ADFs in a variety of immune signaling processes in both plants and animals.

Phosphorylation of ADF and cofilin is indispensable for their functions^9,34,35^. In humans, LIM kinases phosphorylate both cofilin and ADF^36^. In plants, the identification of the ADF kinase(s) relevant to host-pathogen interactions has remained elusive; however, several candidate kinases have been identified^7,8^. Previous work, together with this study, demonstrates that when unphosphorylated, Arabidopsis ADF4 is able to bind ADP-bound F-actin with high affinity and can sever and depolymerize stabilized filaments^13^. Conversely, phosphorylated ADF4 (and cofilin) exhibits a lower affinity for F-actin filaments, and thus is, therefore, considered inactive. Additional in vivo analyses of two members of the Arabidopsis ADF family supports these in vitro models^13,16^.

Herein, we have identified CPK3 as a kinase responsible for the phosho-regulation of ADF4. Based on the sum of the data presented herein, we proposed that ADF4 is a key executor of pathogen-triggered actin reorganization in plants, and that CPK3 is the regulatory kinase controlling ADF4-mediated actin dynamics during immune signaling. As further support for this mechanism, the finding presented herein extend previous work, which demonstrated that independent disruption of two guard cell-expressed CPK genes—CPK3 and CPK6—lead to impairment of ABA- and Ca^2+^-dependent stomatal closure^37^, key signaling processes also associated with the activation and regulation of plant immunity. As entry points for bacterial and fungal plant pathogens, stomatal guard cell gating represents a critical point in immune signaling.

Coupled with work previous work by Mori et al.^37^., our data describe a mechanism linking immune-induced changes in actin cytoskeleton organization to the modulation of stomatal closure. Taken together, these data provide evidence linking actin to additional key signaling processes mediated by CPK, including hormone signaling, pathogen virulence, and immunity.

As a mechanism associating CPK3 with ETI, two observations were key in assigning ETI-based functions. First, we identified CPK3 as a target of AvrPphB, as AvrPphB physically interacts with and destabilizes CPK3. The data presented herein support the hypothesis that this is due to either direct enzymatic cleavage of CPK3 by AvrPphB, or potentially, cleavage of unknown CPK3 interactor(s), which in turn lead to destabilization of CPK3. In either case, our data support a mechanism that describes a previously undefined virulence activity for AvrPphB whereby the reduction of CPK3 results in decreased phosphorylation of ADF4 and less regulatory power to mediate actin remodeling. As a consequence of altered actin cytoskeletal dynamics, immune signaling is abrogated. Interestingly, while we demonstrate that ADF4 phosphorylation inhibits the ADF4-actin interaction, whether such inhibition represents an overall increase in actin filament severing/depolymerization, or F-actin stabilization, remains unanswered.

Taken together, the data presented herein provide a mechanistic understanding of *cpk3-2* susceptibility following *Pst* DC3000-AvrPphB infection, one that is underpinned by pathogen-targeting of the CPK3-ADF4 phospho-switch. This is important, as it demonstrates ADF4 phosphorylation is a key step in the transition from a homeostatic surveillance function of the plant actin cytoskeleton to that of an activated immune signaling platform. In support of this hypothesis, we show that *cpk3-2* mutant plants inoculated with *Pst*-AvrPphB show enhanced susceptibility and attenuation of the HR, compared to WT Col-0, which phenocopies our previous observation of enhanced susceptibility in the *adf4* mutant. Additional *cpk* mutants tested (e.g., *cpk6*, *cpk10*, and *cpk28*) were not susceptible to *Pst* DC3000-AvrPphB. These data provide compelling evidence in support of the hypothesis that *Pst*-AvrPphB targets the host immune system through inhibiting CPK3 functionality, a process required for proper function of ADF4 and organization of the actin cytoskeleton.

## Methods

### Plant genotypes and growth

*Arabidopsis thaliana* plants used in this study includes wild-type (WT) Col-0, the *cpk3-2* mutant (SALK-022862^37^;), the *cpk6* mutant (SALK-025460^37^;), the *cpk28* mutant (CS336535^38^;), the *adf4-2* mutant (SALK-121647^8^;). DNA primers for SALK mutant validation are listed in Supplementary Table 1. Arabidopsis seeds were stratified for 2 d in the dark at 4 °C then sown onto soil. All plants were grown in a BioChambers model FLX-37 walk-in growth chamber (BioChambers, Manitoba, Canada) at 20 °C under long day conditions (16 h of light/8 h of dark) with 60% relative humidity and a light intensity of ~120 μmol photons m^−2 ^s^−1^. For mutant complementation, the *cpk3-2* mutant was transformed via the floral dip method^39^ with an *Agrobacterium tumefaciens* strain, GV3101, harboring the binary vector pEarleygate-203^40^, which contained either the full-length open reading frames (ORF) of *CPK3* and the CPK3 kinase autophosphorylation mutant variant K107M (i.e., CPK3^K107M^). Transgenic plants were selected with glufosinate-ammonium (BASTA, MiliporeSigma, Cat # 45520).

### Bacteria growth

*Escherichia coli* (*E. coli*) and *Agrobacterium tumefaciens* clones were grown on Luria-Bertani (LB) medium containing antibiotics as prescribed by plasmid-borne antibiotic resistance markers. Antibiotics were purchased from GoldBio. *Pseudomonas syringae* pv. *tomato* DC3000 (*Pst* DC3000) strains were grown as previously described^13^. Antibiotics (and final concentrations) used in this study include gentamycin (Cat # G-400-1, 50 μg mL^−1^), kanamycin (Cat # K-120-5, 50 μg mL^−1^), rifampicin (Cat # R-120-1, 100 μg mL^−1^), spectinomycin (Cat # S-140-5, 50 μg mL^−1^).

### In planta bacterial growth assays

*P. syringae* strains were either hand-infiltrated using a 1-mL needleless syringe as previously described^41^, or dip-inoculated as described by Kunkel et al.^39^. For in planta bacterial growth enumeration following hand-infiltration of Arabidopsis leaves, *Pst* DC3000 constructs were inoculated using a 1-mL needleless syringe at a final concentration of 2 × 10^5^ CFU mL^−1^. For dip-inoculation-based assays, *Pst* DC3000 was inoculated at a final concentration of 3 × 10^7^ CFU mL^−1^. Samples were collected at 0- and 72-h post-inoculation (hpi) for hand-infiltration and 96 hpi for dip-inoculation assays. For analysis of the hypersensitive response (HR), leaves from 4-week-old plants were hand-infiltrated with *Pst* DC3000 constructs at a concentration of 10^7^ CFU mL^−1^ using a 1-mL needleless syringe. Leaves were photographed at 20 hpi.

For pathogen-associated molecular pattern (PAMP)-triggered immunity (PTI) priming experiments, 4-week-old Arabidopsis plants were hand-infiltrated with flg22 (1 μM) and incubated at room temperature (ca. 22 °C) for 24 h. After 24 h, flg22-infiltrated leaves were hand-infiltrated with *Pst* DC3000 strains harboring the vector pVSP61. All pathogen-inoculation experiments were performed at least three times with three technical replicates per experiment. A one-way analysis of variance (ANOVA) using GraphPad Software (Prism) was employed for statistical analysis of in planta bacterial growth in the various plant genotypes.

### Cloning and DNA mutagenesis

For protein purification, the open reading frames (ORFs) of *CPK3*, *CPK6*, *CPK28*, *PBS1*, *BIK1*, and *ADF4* were amplified by polymerase chain reaction (PCR) from cDNA, which was reverse-transcribed from total RNA isolated from WT Col-0 and cloned into pENTR/D-TOPO (Invitrogen). The resultant ORF constructs were verified by DNA sequencing (MSU Research Technology Support Facility). The sequence confirmed cDNA constructs of interest were cloned into the bacterial expression vector pDEST17 (Invitrogen) using Gateway recombination (i.e., LR Clonase; Invitrogen). Phosphomimetic and phosphor-null derivatives of ADF4, as well as the catalytically inactive CPK3 variant K107M, were created by Gibson cloning according to the manufacturer’s instructions (New England Biolabs). All DNA primers used for cloning in this study are listed in Supplementary Table 2.

### Protein expression, purification, and kinase assays

Plasmid constructs containing the ORFs of the various CPK and ADF4 derivatives of interest were transformed into OverExpress™ C41 (DE3) *E. coli* competent cells (Lucigen) and the constructs were expressed for 24 h at 14 °C (shaking at 225 rpm). Bacterial cultures were induced with 0.5 mM IPTG (isopropyl β-D-1-thiogalactopyranoside; MiliporeSigma, Cat # I6758) at a bacterial culture density of OD_600nm_ = 0.8 (ca. 6.4 × 10^8^ CFU mL^−1^). Proteins were batch purified using Ni-NTA resin (Qiagen) according to the manufacturer’s recommendations. Eluted proteins were further purified by fast protein liquid chromatography (FPLC) using a Superdex 200 10/300 GL column (GE Healthcare) under physiological conditions (0.05 M NaH_2_PO_4_ and 0.15 M NaCl, pH 7.5).

Protein phosphorylation assays reactions consisted of 5 μg of substrate, 0.5 μg of kinase, 0.5 μL of radiolabelled ATP [γ-^32^P] (Perkin Elmer), and 4 μL of 5x buffer (0.1 M Tris-HCl, pH 7.5, 50 mM MgCl_2_, 1 mM CaCl_2_, 5 mM DTT, 1 complete, mini, EDTA-free protease inhibitor cocktail tablet (MiliporeSigma, Cat # 11836170001; 1 tablet/50 mL homogenization buffer) in a total final volume of 20 μL. Reactions were incubated for 1 h at 30 °C and stopped by the addition of 5x Laemmli sample buffer. Kinase reactions were resolved by 4–12% sodium dodecyl sulphate–polyacrylamide gel electrophoresis ((SDS-PAGE) (NuPAGE, Invitrogen)), and gels were stained with CBB for 1 h at room temperature (ca. 22 °C), followed by destaining in clearing buffer (10% acetic acid, 20% methanol). Destained gels were dried under a vacuum (Slab Gel Dryer SE1160, Hoefer). Reactions were visualized by detection on X-ray film. Negative control reactions were run in parallel and consisted of each of the aforementioned components yet contained either no kinase or no substrate.

### RNA isolation and quantitative real-time PCR

For mRNA expression analyses, 4-week-old Arabidopsis leaves were syringe-infiltrated with flg22 (1 μM) or *Pst* DC3000 strains [3 × 10^7^ CFU mL^−1^] using a 1-mL needleless syringe and incubated at 22 °C. For analysis of mRNA accumulation following flg22 treatment, samples were collected at 0, 30 min, 1 h, and 3 h post-inoculation. For *Pst* DC3000 infection, leaf samples were collected at 0, 6, and 12 h after inoculation. Total RNA was extracted from samples using the RNeasy Plant Mini kit (Qiagen). One microgram of total RNA was used for first-strand cDNA synthesis using the Maxima H Minus First-Strand cDNA synthesis kit (ThermoFisher Scientific). All DNA primers used for quantitative real-time PCR (qPCR) are listed in Supplementary Table 3. Hot Start SYBR Master Mix 2x (USB Affymetrix) was used to perform qRT-PCR, using an Applied Biosystems 7500 Fast Real-Time PCR System (ThermoFisher Scientific). Amplification of *PP2A* was used as an internal control. Fold expression was calculated as previously described^13^. All mRNA expression data was analyzed using GraphPad Software (Prism). Expression values are represented as mean ± standard error of the mean (SEM). Statistical analysis was evaluated using a two-way ANOVA, followed by the Bonferroni post-test as compared to WT Col-0. *p-*values ≤ 0.05 were considered significant, where *p-value < 0.05, **p-value < 0.01, and ***p-value < 0.005.

### MAPK phosphorylation assays

For MAPK phosphorylation assays, 14-day-old Arabidopsis seedlings were stimulated with flg22 (100 nM) by floating a solution of flg22 peptide (dissolved in water) onto sterile 14-day-old seedlings grown on ½ MS medium (0.7% agar plates). Seedlings were elicited with flg22 for 0, 10, 20, 30, and 60 min at room temperature (ca. 22 °C). At each time point, 15 seedlings were harvested, flash-frozen in liquid nitrogen, and stored at −80 °C. Samples were processed as previously described^13,41^. Samples (30 μg total protein) were resolved by SDS-PAGE and western blot analysis of phosho-MPK3/6 was performed using an anti-pTEpY antibody (Cell Signaling Technology, Cat # 9101S).

### ROS assay

PAMP-induced reactive oxygen species (ROS burst) was measured following a previously reported method^42^, with slight modification. Briefly, leaf disks from 4-week-old Arabidopsis plants were harvested using a 4-mm biopsy punch (Miltex) and floated onto 100 μL of sterile dH_2_O in a 96 well-plate, overnight, without motion. After the overnight incubation, the water was carefully removed using a multichannel pipette, and 200 μL of a working solution containing 100 nM flg22, 10  μg mL^−1^HRP (horse radish peroxidase, MiliporeSigma, Cat # P8375), and 30 μg mL^−1^ luminol (MiliporeSigma, Cat # A8511) was added to each well. After addition of the working solution, the plate was immediately moved to a microplate reader (SpectraMax-L, Molecular Devices) for measurement of luminescence. Measurements were taken over a 1 h time course, with a step of 2 min. Three biological repeats were performed, each with similar results. For each assay, four leaf disks from four individual plants were used. All reaction steps were conducted at room temperature.

### Mass spectroscopy analysis

To identify phosphorylated residues in ADF4, 20 μg of purified proteins was incubated, individually, with 2 μg of purified CPK3, BIK1, and PBS1 in kinase buffer for 1 h. Phosphorylation reactions were separated by SDS-PAGE (15% Tris-Bis), and resolved gels were stained with CBB. The stained protein bands corresponding to ADF4 or PBS1^K115N^ were excised and was subjected to in-gel digestion according to the method of Shevchenko et al.^43^, with slight modification. Briefly, excised gel bands were dehydrated in 100% acetonitrile (ACN) and incubated with 10 mM DTT in 100 mM ammonium bicarbonate (pH 8), at 56 °C for 45 min. After 45 min, the samples were dehydrated again and incubated in the dark with 50 mM iodoacetamide in 100 mM ammonium bicarbonate for 20 min. Gel bands were then washed with ammonium bicarbonate and dehydrated again. Sequencing grade modified trypsin was prepared to 0.01 μg μL^−1^ in 50 mM ammonium bicarbonate and 50 μL of trypsin was added to each sample such that the gel was completely submerged. Samples were incubated at overnight at 37 °C. Peptides were extracted from the gel by water bath sonication in a solution of 60% ACN/1% TCA and vacuum dried to ~2 μL. Peptide samples were then re-suspended in 2% ACN/0.1%TFA to 20 μL. From this, 5 μL was injected onto a Thermo Acclaim PepMap RSLC 0.075 mm × 20 mm C18 trapping column and washed for ~5 min using a Thermo EASYnLC. Peptides were then eluted onto a Thermo Acclaim PepMap RSLC 0.075 mm × 500 mm C18 analytical column over 35 min with a gradient of 2% B to 40% B over 24 min, ramping to 100% B at 25 min and held at 100% B for the duration of the run (Buffer A = 99.9% water/0.1% formic acid, Buffer B = 80% ACN/0.1% formic acid/19.9% water).

Eluted peptides were sprayed into a ThermoFisher Q-Exactive HF-X mass spectrometer using a FlexSpray spray ion source. Survey scans were taken in the Orbitrap (45,000 resolution, determined at *m*/*z* 200) and the top twenty ions in each survey scan were then subjected to automatic higher energy collision induced dissociation (HCD) with fragment spectra acquired at 7500x resolution. The resultant MS/MS spectra were converted to peak lists using Mascot Distiller (ver. 2.7.0; www.matrixscience.com) and the output was searched against all entries in the TAIR (ver. 10) protein sequence database (downloaded from The Arabidopsis Information Network; www.arabidopsis.org). All entries in the UniProt *E. coli* protein sequence database (downloaded from www.uniprot.org, 2017-11-01), appended with common laboratory contaminants (downloaded from www.thegpm.org, cRAP project), were scanned using the Mascot searching algorithm (ver. 2.6). The Mascot output was analyzed using Scaffold Q + S (ver. 4.8.4; www.proteomesoftware.com) to probabilistically validate protein identifications. Assignments validated using the Scaffold 1% FDR confidence filter were considered true.

### Construction of gateway vectors for Agrobacterium and protoplast transformation

Construction of the Agrobacterium subcellular localization gateway vectors pBGWB was as follows: pSAT5(A)-DEST-c(175-end)-EYFP-N1 (pE3132) was purchased from TAIR (Cat #CD3-1096). To restore the *Xba*I cleavage site from DAM methylation, the sequence between, and including, *Xba*I and *Eco*RI restriction sites was amplified by PCR. After PCR amplification, the PCR products were resolved by gel electrophoresis, digested with *Xba*I and *Eco*RI, and ligated into pNeo1096.

Construction of the protoplast gateway vectors p5GWH was performed as follows: an *Apa*I-HA-*Xba*I and an *Apa*I-Myc-*Xba*I dsDNA oligo with sticky ends was generated by annealing two pairs of corresponding DNA primers and ligated into pNeo1096 to generate p5GWH (for HA tag) in place of the original cEYFP. p5GWH::ADF4 was constructed by LR cloning. All DNA primers are listed in Supplementary Table 4.

### *Agrobacterium*-mediated transient expression and co-immunoprecipitation assays

Sequence confirmed binary vector constructs harboring the ORF of *ADF4* (including various derivative mutants) were electroporated into *A. tumefaciens* strains GV3101 and maintained on LB plates containing rifampicin (50 μg mL^−1^), kanamycin (100 μg mL^−1^), gentamycin (50 μg mL^−1^). For expression of all *ADF4* constructs, the cDNAs were cloned into the binary vector pEarleygate-202^40^.

For transient expression in *Nicotiana benthamiana*, the method of Shimono et al.^15^ was used, with slight modifications. *A. tumefaciens* GV3101 strains harboring expression constructs of interest were pre-incubated at room temperature (ca. 22 °C) in induction media (10 mM MES [pH 5.6], 10 mM MgCl_2_, 150 mM acetosyringone [MiliporeSigma, Cat # D134406]) for 2 h before hand-infiltration into 5-week-old *N. benthamiana* leaves using a 1-mL needleless syringe. Inoculated plants were kept at 22 °C for 40 h (12 h of light/12 h of dark), after which time 18 leaf discs (1 cm^2^/each) were harvested and flash-frozen into liquid nitrogen. For co-immunprecipitation analyses, leaf disks were processed as previously described^41^, with slight modifications. Total protein extracts were isolated by grinding leaf discs in liquid nitrogen into a fine powder, and the powder was transferred to a chilled (4 °C) mortar and further homogenized in homogenization buffer (50 mM HEPES [pH 7.5], 50 mM NaCl, 10 mM ethylenediaminetetraacetic acid [EDTA], 0.2% triton X-100, and 1 cOmplete, mini, EDTA-free protease inhibitor cocktail tablet [1 tablet/50 mL homogenization buffer]). Homogenized samples were centrifuged at 4 °C at 15,000 × *g* for 15 min, and 1 mL of the resultant supernatant was transferred to a sterile 2-mL microcentrifuge tube. Samples were tumbled end-over-end at 4 °C for 1.5 h with either 5 μL of anti-Flag M2 monoclonal antibody (MilliporeSigma, cat # F3165) or 10uL of anti-actin monoclonal antibody (MilliporeSigma, cat # A3853). After 1.5 h, 50 μL of protein-G sepherose-4 fast flow (GE Healthcare), pre-washed [50 mM HEPES (pH 7.5), 50 mM NaCl, 10 mM EDTA, 0.1% triton X-100, and 1 cOmplete mini EDTA-free protease inhibitor cocktail tablet] (was added to each tube and tumbled end-over-end for 4 h at 4 °C. Input samples were incubated in parallel; these samples did not receive antibodies or protein-G sepharose. After 4 h, samples were centrifuged at 15,000 × *g* for 15 s, the supernatant discarded, and the pellets were washed 3 times with wash buffer [50 mM HEPES (pH 7.5), 50 mM NaCl, 10 mM EDTA, 0.1% triton X-100]. For SDS-PAGE (10% Bis-Tris) analysis, 25 μL of the input sample, 10 μL of the immunoprecipitation sample, and 20 μL of the co-immunprecipitation samples were separated by electrophoresis, and resolved samples were transferred to nitrocellulose (BioRad, Cat # 1620115). Signals were detected using the Super Signal West Pico Chemiluminescent Substrate (ThermoFisher, Cat # 34577), with anti-Flag M2-peroxidase (MilliporeSigma, Cat # A8592).

### ADF4-actin co-localization

*ADF4* (including various derivative mutants) and fABD2 were cloned in to *pGWB555*^44^ and *pART27*^10^, respectively, and the *Agrobacterium*-mediated transient expression in *N. benthamiana* was conducted following the procedure stated above. Leaf disks from 48 hpi samples were observed and photographed using a 60x/1.42 PlanApo N objective and a 100x/1.40 objective on an Olympus FV1000D.

### Mesophyll cell protoplast preparation and transformation

For protoplast preparation, 5-week-old Arabidopsis leaves were harvested following previously described methods^45^. Approximately 10^5^ protoplasts were transformed with 10 μg of plasmid DNA containing various expression constructs of interest. Following transformation, protoplasts were incubated at room temperature under weak light for 14 h, after which time samples were harvested and processed for analysis.

### Two-dimensional gel electrophoresis

Five-week-old Arabidopsis leaves were harvested from both WT Col-0 and the *cpk3-2* mutant for protoplast preparation as described above. For 2DGE, 10^5^ protoplasts were transformed with 10 μg of p5GWH::ADF4, and after 14 h incubation at room temperature. 200 μg of HA-ADF4 protein was isolated for both WT Col-0 and *cpk3-2* mutant transformed protoplasts. Total protein extracts were precipitated with chloroform:methanol (1:4) and reconstituted in urea buffer [7 M urea, 2 M thiourea, 2% CHAPS, 2% ASA-14, 50 mM DTT, 0.2% Biolyte ampholytes and 0.1% bromophenol blue). Isoelectric focusing and SDS-PAGE were conducted according to the manufacturer’s instructions (BioRad). Immunoblot analysis was performed described above. The detection of HA-ADF4 was performed by western blot analysis using anti-HA-HRP antibodies (MilliporeSigma, Cat # 12013819001).

### Stomatal closure assays

Four-week-old Arabidopsis plants were used for stomatal closure assays. In brief, leaves were floated in MES/KOH buffer [50 mM KCl, 10 μM CaCl_2_, 0.01% Tween 20, 10 mM MES (pH 6.15)] and incubated at 22 °C in a Percival incubator with constant light for 3 h. Once stomatal apertures stabilized, leaves were incubated under the same conditions, with mock or 10 μM flg22 for 1 h. For time course analysis of stomata closure, 10-to-13-day-old Arabidopsis seedlings were used. Plants were treated with 1 μM flg22 or *Pst* DC3000 by dip-inoculation. Images of lower epidermal leaf surfaces were observed, and images were collected using an Olympus FV1000D fluorescent confocal microscope (405 nm laser to measure autofluorescence of the cell walls from the guard cell, and a 488 nm laser to measure the GFP-fABD2 signal in transgenic plants). The ratio of stomatal aperture (width/length) was analyzed using ImageJ.

### G-actin/F-actin in vivo assay and actin disruption assay

To monitor the ratio of G-actin versus F-actin in WT Col-0 and the *cpk3* mutant after the activation of immune signaling, a commercially available quantification kit was used, and G-actin/F-actin in vivo assays were performed according the manufacturer’s instructions with slight modification. Isolate of G-actin and F-actin from Arabidopsis leaves was performed using G-actin/F-actin in vivo assay kit (Cytoskeleton, Inc., Cat # BK037). G-actin and F-actin fractions were separated alongside 500, 200, and 75 ng of G-actin standards (bovine cardiac muscle actin, Cytoskeleton, Inc., cat # AD99-A) on an SDS polyacrylamide gel (4–12% Bis-Tris gel, Invitrogen, Carlsbad, CA). Relative actin quantification was determined by western blot analysis using monoclonal anti-actin (plant) antibody (MiliporeSigma, CAT # A0480-25 μL).

To monitor F-actin disruption, AvrPphB-6xHis and AvrPphB^C98S^-6xHis were expressed in *E. coli* (C41,DE3) and purified as described above for CPK3 and ADF4. Actin disruption and polymerization assays were performed according to the manufacturer’s instructions (Cytoskeleton Inc, Cat # BK013). G- and F-actin fractions were isolated by ultracentrifugation (150,000 × *g*) and proteins were separated by SDS polyacrylamide gel electrophoresis (4–12% Bis-Tris gel, Invitrogen). Relative actin quantification was determined by CBB staining using know amounts of commercially available protein standards (Cytoskeleton Inc).

### Confocal microscopy and quantitative evaluation of actin filament organization

To evaluate actin organization including density, bundling and orientation analyses were performed according to previously described methods^46–48^. All image collection and quantitative analyses were performed as double-blind experiments, in biological triplicate. Actin filament analyses were performed on confocal microscopy imaged fields of epidermal pavement cells and guard cells. Images were collected using laser confocal scanning microscopy by obtaining 27 z-series sections at 0.5 µm intervals. Laser confocal scanning microscopy was performed using a 60x/1.42 PlanApo N objective on an Olympus FV1000D. Serial optical sections were projected with maximum intensity and analyzed in ImageJ using algorithms previously described^46–48^. Gaussian blur and bandpass filters were applied to stack images before generating projection images for density analysis. Statistical analyses were performed using the Mann–Whitney *U*-test with GraphPad software (Prism). Density, also referred to as “Percent Occupancy”, indicates the relative abundance of actin filament signal within a cell. Skewness is an indirect measure of the degree of actin filament bundling. Parallelness indicates variation in actin cytoskeletal orientation^47,48^.

### Immunocomplex kinase assays

Protoplasts isolated from *cpk3-2/35S::CPK3* were digested with 0.5 mL of immunoprecipitation buffer (50 mM Tris·HCl, pH 7.5, 150 mM NaCl, 5 mM EDTA, 1 mM DTT, 1% Triton, and 1 cOmplete, mini, EDTA-free protease inhibitor cocktail tablet (1 tablet/50 mL immunoprecipitated buffer). After centrifugation at 15,000 × *g* for 10 min at 4 °C, the supernatant was incubated with anti-Myc antibody (AbCam, Cat# ab32) for 2 h and then gently tumbled with protein-G–agarose beads for 2 h at 4 °C. The beads were collected and washed twice with immunoprecipitation buffer and once with kinase buffer (20 mM Tris·HCl, pH 7.5, 10 mM MgCl_2_, 200 nM CaCl_2_, 1 mM DTT and cOmplete mini, EDTA-free protease inhibitor). The washed beads incubated in 20 μL of kinase buffer with 1 μg of myelin basic protein (MBP, MiliporeSigma, Cat # M1891) and 0.5 μL of ATP [γ-^32^P] at 30 °C for 1 h with gentle shaking. Kinase reactions were analyzed by 4–12% SDS-PAGE (NuPAGE, Invitrogen).

### Reporting summary

Further information on research design is available in the Nature Research Reporting Summary linked to this article.

## Supplementary information

Supplementary Information

Reporting Summary

## Data Availability

The authors declare that all data supporting the findings of this study are included in the manuscript and its supplementary files or are available from the corresponding author upon request. Source data are provided with this paper.

## Source data

Source Data
