# Supplementary material for: Arabidopsis calcium-dependent protein kinase 3 regulates actin cytoskeleton organization and immunity
Source: Nat Commun. 2020 Dec 4;11:6234. doi: 10.1038/s41467-020-20007-4 (PMC7718926; doi:10.1038/s41467-020-20007-4)
Supplement: Supplementary file 2 — Reporting Summary [file 41467_2020_20007_MOESM2_ESM.pdf]

nature research

Corresponding author(s): Brad Day

Last updated by author(s): Nov 3, 2020

Reporting Summary

Nature Research wishes to improve the reproducibility of the work that we publish. This form provides structure for consistency and transparency in reporting. For further information on Nature Research policies, see Authors & Referees and the Editorial Policy Checklist .

Statistics

For all statistical analyses, confirm that the following items are present in the figure legend, table legend, main text, or Methods section.

n/a	Confirmed	
☐	☒	The exact sample size (*n*) for each experimental group/condition, given as a discrete number and unit of measurement
☐	☒	A statement on whether measurements were taken from distinct samples or whether the same sample was measured repeatedly
☐	☒	The statistical test(s) used AND whether they are one- or two-sided<br>*Only common tests should be described solely by name; describe more complex techniques in the Methods section.*
☐	☒	A description of all covariates tested
☐	☒	A description of any assumptions or corrections, such as tests of normality and adjustment for multiple comparisons
☐	☒	A full description of the statistical parameters including central tendency (e.g. means) or other basic estimates (e.g. regression coefficient) AND variation (e.g. standard deviation) or associated estimates of uncertainty (e.g. confidence intervals)
☐	☒	For null hypothesis testing, the test statistic (e.g. *F*, *t*, *r*) with confidence intervals, effect sizes, degrees of freedom and *P* value noted<br>*Give P values as exact values whenever suitable.*
☒	☐	For Bayesian analysis, information on the choice of priors and Markov chain Monte Carlo settings
☒	☐	For hierarchical and complex designs, identification of the appropriate level for tests and full reporting of outcomes
☒	☐	Estimates of effect sizes (e.g. Cohen's *d*, Pearson's *r*), indicating how they were calculated

Our web collection on statistics for biologists contains articles on many of the points above.

Software and code

Policy information about availability of computer code

Data collection	No software was used for data collection
Data analysis	1, Graph Pad Prism 9 was used to analyze all statistics for quantitative results.<br>2, Image J was used to analyze the intensity for bands generated by western blots or radiation labelling.<br>3, Image J was used for the analysis of actin filament organization (bundling and density) using a custom, publically-available script, as described in our previous publication: Lu, Y-J., and Day, B. (2017). Quantitative evaluation of plant actin cytoskeletal parameters during immune activation. In: Methods in Mol. Biol. Shan, L., and He, P., Eds. DOI:10.1007/978-1-4939-6859-6856. This method is cited in the current manuscript.

For manuscripts utilizing custom algorithms or software that are central to the research but not yet described in published literature, software must be made available to editors/reviewers. We strongly encourage code deposition in a community repository (e.g. GitHub). See the Nature Research guidelines for submitting code & software for further information.

Data

Policy information about availability of data

All manuscripts must include a data availability statement. This statement should provide the following information, where applicable:

- Accession codes, unique identifiers, or web links for publicly available datasets
- A list of figures that have associated raw data
- A description of any restrictions on data availability

All raw data for this research are included in source data Excel file.

Field-specific reporting

Please select the one below that is the best fit for your research. If you are not sure, read the appropriate sections before making your selection.

[x] Life sciences [ ] Behavioural & social sciences [ ] Ecological, evolutionary & environmental sciences

For a reference copy of the document with all sections, see nature.com/documents/nr-reporting-summary-flat.pdf

Life sciences study design

All studies must disclose on these points even when the disclosure is negative.

Sample size	1, For bacterial growth curve assays, 3 leaves from least 3 independent plants were collected.<br>2, For quantitative actin analysis and stomatal aperture, we attempted to reach the maximum sample sizes as we could in each biological repeat. For each biological repeat, a minimum of 30 images per sample was evaluated.
Data exclusions	No data points were excluded from any analyses described in this study. No outlier tests were employed to exclude data.
Replication	Experiments were repeated at least 3 times, often containing multiple technical replicates within each. For kinase assays, experiments were repeated multiple times, using independent protein purification samples. All plant-based assays were repeated at least 3 times, using independently grown plants; that is, plants that reached 4-5-weeks-old at different times.
Randomization	With the exception of minor randomization of plant growth (to minimize growth effects due to placement in the plant growth chamber), no randomization was undertaken. All plants used in this study were grown under the same conditions and in the same growth chamber.
Blinding	No blinding was utilized for the experiments described in this manuscript.

Reporting for specific materials, systems and methods

We require information from authors about some types of materials, experimental systems and methods used in many studies. Here, indicate whether each material, system or method listed is relevant to your study. If you are not sure if a list item applies to your research, read the appropriate section before selecting a response.

Materials & experimental systems

n/a	Involved in the study	
[ ]	[x]	Antibodies
[x]	[ ]	Eukaryotic cell lines
[x]	[ ]	Palaeontology
[x]	[ ]	Animals and other organisms
[x]	[ ]	Human research participants
[x]	[ ]	Clinical data

Methods

n/a	Involved in the study	
[x]	[ ]	ChIP-seq
[x]	[ ]	Flow cytometry
[x]	[ ]	MRI-based neuroimaging

Antibodies

Antibodies used	Name / Clone (Vendor, Cat#)<br><br>Phospho-p44/42 MAPK (Erk1/2) (Thr 202/Tyr204) antibody / Rabbit Polyclonal (Cell Signaling Technology, Cat # 9101S)<br>Anti-rabbit IgG, HRP-linked Antibody / Goat Secondary (Cell Signaling Technology, Cat # 7074)<br>Anti-Myc tag antibody [9E10]/ Mouse Monoclonal (AbCam, Cat # ab32)<br>Anti-HA-Peroxidase [3F10] / Rat Monoclonal (Millipore Sigma, Cat # 12013819001)<br>Anti-Actin (plant) antibody [10-B3] / Mouse Monoclonal (Millipore Sigma, Cat # A0480)<br>Anti-MYC tag antibody (HRP) / Rabbit Polyclonal (Abcam, Cat # ab1326)<br>Anti-Flag M2 antibody / Mouse Monoclonal (Millipore Sigma, Cat # F3165)<br>Anti-Flag M2-Peroxidase (HRP) antibody / Mouse Monoclonal (Millipore Sigma, Cat # A8592)
Validation	The validation information for all antibodies is available from vendor's website for each antibody.
