# Supplementary material for: Arabidopsis calcium-dependent protein kinase 3 regulates actin cytoskeleton organization and immunity
Source: Nat Commun. 2020 Dec 4;11:6234. doi: 10.1038/s41467-020-20007-4 (PMC7718926; doi:10.1038/s41467-020-20007-4)
Supplement: Supplementary file 1 — Supplementary Information [file 41467_2020_20007_MOESM1_ESM.docx]

| **Gene** | **Forward Primer** | **Reverse Primer** |
| --- | --- | --- |
| *cpk3* | 5´-AAAAGGATCCGGGCCCATGGGCCACAGACACAGCAAGTCCAAATCCTCCG-3´ | 5´-TTTTGTCGACCTAGCGGCCGCACATTCTGCGTCGGTTTGGCACCAATTCTGGATTTCCC-3´ |
| *cpk6* | 5´-CTCGCAACTAACGCTTACCTG-3´ | 5´-CTCCATTTCATCGTCTTCTCG-3´ |
| *cpk28* | 5´-CCACTAACCCATCAGTGTTGC-3´ | 5´-AAGGTTCCATGACATTGTTG-3´ |
| *LBb1.3* | 5´-ATTTTGCCGATTTCGGAAC-3´ |  |
| *GABI-KAT* | 5´-ATATTGACCATCATACTCATTGC-3´ | 5´-GTGGATTGATGTGATATCTCC-3´ |

Supplementary Table 1. DNA primers used for Arabidopsis mutant genotyping.

| **Clone** | **Forward Primer** | **Reverse Primer** |
| --- | --- | --- |
| *ADF4* | 5´-caccATGGCTAATGCTGCGTCA-3´ | 5´- TTAGTTGACGCGGCTTTTCAAAAAC-3´ |
| *ADF4^S6A^* | 5´-caccATGGCTAATGCTGCGGCAGG-3´ | 5´-CCTGCCGCAGCATTAGCCAT-3´ |
| *ADF4^S6D^* | 5´-caccATGGCTAATGCTGCGGACGGAATGG-3´ |  |
| *ADF4^S105A^* | 5´- TATGCGGCCTCTAAGGACAGGTTCAAG-3´ | 5´- TTAGAGGCCGCATAGATCATCTTGC-3´ |
| *ADF4^S105D^* | 5´-ATGATCTATGCGGACTCTAAGGACAGGTTCAAGCGTGA-3´ | 5´-GAACCTGTCCTTAGAGTCCGCATAGATCATCTTGCTTCTC-3´ |
| *ADF4^S106A^* | 5´-GCGAGCGCTAAGGACAGGTTCAAGC -3´ | 5´- GTCCTTAGCGCTCGCATAGATCATCTTGC-3´ |
| *ADF4^S106D^* | 5´-ATGATCTATGCGAGCGATAAGGACAGGTTCAAGCGTGA-3´ | 5´-GAACCTGTCCTTATCGCTCGCATAGATCATCTTGCTTCTC-3´ |
| *ADF4^S105/106A^* | 5´-CGGCCGCTAAGGACAGGTTC-3´ | 5´- CCTTAGCGGCCGCATAGATCATCTTG -3´ |
| *ADF4^S105/106D^* | 5´-ATGATCTATGCGGACGATAAGGACAGGTTCAAGCGTGA-3´ | 5´-GAACCTGTCCTTATCGTCCGCATAGATCATCTTGCTTCTC-3´ |
| *CPK3-*Gateway_SalI | 5´-caccGTCGACATGGGCCACAGACACAGC |  |
| *CPK3-*cMyc_Stop_SacI |  | 5´-GAGCTCCTACAGATCCTCTTCTGAGATGAGTTTTTGTTCCATTCTGCGTCGGTTTG-3´ |
| *CPK3^K107M^* | 5´-TCGCTTGCATGTCAATCCCTAC-3´ | 5´- GTAGGGATTGACATGCAAGCGA-3´ |
| *CPK6-*Gateway_SalI | 5´-caccGTCGACATGGGCAATTCATGTCGTGGTT-3´ |  |
| *CPK6-*cMyc_Stop_SacI |  | 5´-GAGCTCCTACAGATCCTCTTCTGAGATGAGTTTTTGTTCCACATCTCTCATGCTGATG-3´ |
| *CPK28-*Gateway_SacI | 5´-caccGAGCTCATGGGTGTCTGTTTCTCCGCCATT-3´ |  |
| *CPK28-* cMyc_Stop_SacI |  | 5´-GAGCTCCTACAGATCCTCTTCTGAGATGAGTTTTTGTTCTCGAAGATTCCTGTGACCT-3´ |
| *AvrPphB-*Gateway | 5´-caccATGAAAATAGGTACGCAGGCCACC-3′ | 5´-AACTCTAAACTCGTTTACGC-3′ |
| *AvrPphB-*Gateway-HA | 5´-caccATGAAAATAGGTACGCAGGCCACC-3´ | 5´-TTATCAAGCGTAATCTGGAACATCGTATGGGTACGAAACTCTAAACTCGTTTACGC-3´ |
| *PBS1-*Gateway | 5´-caccATGGGTTGTTTCTCGTGTTTT-3´ | 5´-CTACCCGGTACTGTTGCTC-3´ |
| *BIK1-*Gateway | 5´-caccATGGGTTCTTGCTTCAGTTCCTACACAAGGTGCCTGCCAA-3´ | 5´- CTACACAAGGTGCCTGCCAA -3´ |

Supplementary Table 2. DNA primers used for the cloning of various constructs described in this study. Lower case “cacc” denotes the added complementary base pairs for GTCC overhang for ligation into each pENTR/D-TOPO vector. Underlined sequences denote specific restriction enzyme recognition sites added to the DNA constructs to facilitate cloning.

| **Gene** | **Forward Primer** | **Reverse Primer** |
| --- | --- | --- |
| *PP2A* | 5´-GGTTACAAGACAAGGTTCACTC-3´ | 5´-CATTCAGGACCAAACTCTTCAG-3´ |
| *CPK3* | 5´-TGAGTTTGGTCGTGAGCTTG-3´ | 5´- AGCGACCTGTTGTTTCGTCT-3´ |
| *FRK1* | 5´-CGGTCAGATTTCAACAGTTGTC-3´ | 5´-AATAGCAGGTTGGCCTGTAATC-3´ |
| *PR-1* | 5´-ACACGTGCAATGGAGTTTGTGG-3´ | 5´-TTGGCACATCCGAGTCTCACTG-3´ |
| *WRKY70* | 5´-TCTCCGTTCTTGATACCTTCG-3´ | 5´-CGTCGTTATCACATGAAGCATT-3´ |
| *WRKY46* | 5´-CGTGCATCTGTAATATGCTCTAGG-3´ | 5´-GATGATGGTCACTGCTGGAG-3´ |

Supplementary Table 3. DNA primers used for quantitative real-time PCR (qRT-PCR) analysis.

| **Vector name** | **Primer Purpose** | **Primer name** | **Sequence** |
| --- | --- | --- | --- |
| pNeo1096 | To remove DAM methylation site | RealSATP1-XbaI-R | 5′-GACTCTAGATTAGGTGGATCTTCACTTGTAC-3′ |
|  | To remove DAM methylation site | XbaI-RealSATP2-F | 5′-TAATCTAGAGTCCGCAAAAATCACCAGTC-3′ |
|  | To remove DAM methylation site | EcoRI-RealpSATP1-F | 5′-ATCCGGAATTCCGTATGGCA-3′ |
|  | To remove DAM methylation site | EcoRI-RealSATP2-R | 5′-ATACGGAATTCCGGATGAGCA-3′ |
|  | Sequencing | Test-pSAT-1 | 5′-GCCGTAATATCCAGCTGAACG-3′ |
|  | Sequencing | Test-pSAT-2 | 5′-AATCTCATGACCAAAATCCC-3′ |
| p5GWH | Add HA epitope tag | ApaI-HA-XbaI-stop-R | 5′-CTAGATCAAGCGTAATCTGGAACATCGTATGGGTAGGGCC-3′ |
|  | Add HA epitope tag | ApaI-HA-XbaI-stop-F | 5′-CTACCCATACGATGTTCCAGATTACGCTTGAT-3′ |

Supplementary Table 4. DNA primers for construction of protein expression constructs for *Agrobacterium* and protoplast transformation experiments.

**Supplementary Figures**

**Supplementary Figure 1.** Coomassie brilliant blue (CBB) stained gels shows equivalent sample loading of *in vitro* kinase reactions. **a** corresponds with Fig.1a; **b** corresponds to Fig. 1b; **c** corresponds to Fig.1c; **d** corresponds to Fig.1d. Experiments were repeated 3 times.

**Supplementary Figure 2.** Mutation of lysine at position 107 to methionine (i.e., K107M) in CPK3 abolishes auto-phosphorylation activity. **a** Phosphorylation of MBP by CPK3, CPK6, and CPK28. **b** The auto-phosphorylation activity of CPK3 requires lysine at position 107. **c** CPK3^K107M^ does not phosphorylate ADF4 and ADF4 does not exhibit a kinase activity associated with auto-phosphorylation. The lower panels (i.e., Coomassie brilliant blue stained gels) shows equivalent sample loading of the reactions. 2.5 μg of substrate and 0.5 μg of kinases, produced in *E. coli*, were used in each reaction. MBP, myelin basic protein. Experiments were repeated 3 times.

**Supplementary Figure 3.** *In silico* predictions and mass spectroscopy analysis of ADF4 phosphorylation by CPK3. **a** Amino acid sequence alignment of predicted serine phosphorylation sites in ADF4, as determined by NetPhos 2.0 (http://www.cbs.dtu.dk/services/NetPhos-2.0/). ‘+’ indicates positive prediction of phosphorylation. **b** Mass spectra and identification of ADF4 serine^105^ phosphorylation by CPK3. **c** Mass spectra and identification of ADF4 serine^106^ phosphorylation by CPK3.

**Supplementary Figure 4.** Mass spectroscopy analysis of ADF4 phosphorylation by PBS1 and BIK1. Mass spectra of ADF4 kinase assay with **a** PBS1 and **b** BIK1. No phosphorylation sites were identified in either kinase assay, with 100% coverage of the sequence of ADF4. Amino acid residues highlighted in yellow illustrates coverage. Amino acids highlighted in green indicate amino acid modifications independent of phosphorylation (e.g., oxidation).

**Supplementary Figure 5. a** In planta co-immunoprecipitation of ADF4 phosphomimic and phosphorylation-null derivatives with anti-actin antibody. Co-immunoprecipitation experiments were performed using *Agrobacterium* transient expression (40 h) in *N. benthamiana*. Total protein extracts containing Flag-epitope-tagged ADF4 constructs were immunoprecipitated using a polyclonal antibody to plant actin and the resultant pull downs were denatured and separated by SDS-PAGE, followed by western blot analysis. These experiments were performed twice with similar results. **b** In vivo fluorescence microscopy localization of ADF4 phosphorylation derivatives and ACT7. Constructs were co-expressed in *N. benthamiana* as C-terminal mTagBFP fusion proteins for ADF4 variants and as a C-terminal mTagYFP fusion protein for ACT7 using *A. tumefaciens*-mediated transient expression. Arrows are shown to indicate co-localization of the fluorescence signal with F-actin. Scale bar = 10 μm. All experiments were performed three times with similar results and 30 images were collected in total for each co-expressed construct.

**Supplementary Figure 6.** Quantitative real-time PCR analysis of *CPK3* mRNA expression in WT Col-0. Analysis of defense and immune-related mRNA expression in Arabidopsis lines following flg22, *Pst* DC3000, *Pst* DC3000-AvrPphB, and *Pst* DC3000-AvrPphB^C98S^ infiltration. Transcript levels of *CPK3* were quantified by qPCR in WT Col-0. mRNA levels were normalized against the endogenous expression of protein phosphatase 2A (*PP2A*). Error bars represent means ± SD from two technical replicates of three independent biological repeats (*n* = 6). Letters indicate statistically significant differences, as determined by a one-way ANOVA (*p* value < 0.05; ns, not significant).

**Supplementary Figure 7.** Analysis of PTI-associated responses in the *cpk3-2* mutant. The *cpk3-2* mutant, including the *CPK3* complementation lines, show WT levels of **a** MAPK phosphorylation and **b** ROS burst activity in response to flg22 treatment. Error bars represent means ± SD from four technical replicates of three independent biological repeats (*n* = 12). MAPK experiment was repeated 3 times.

**Supplementary Figure 8.** CPK3 is not required for PTI-induced bacterial resistance priming. **a** Four-week-old plants (WT Col-0 and the *cpk3-2* mutant) were syringe-infiltrated with 10 μM flg22 peptide 24 h before the same leaves were syringe-infiltrated with *Pst* DC3000 (10^7^ CFU/ml). Plants were incubated at 22 °C (12 h light/12 h dark) for 2 days and bacterial growth was enumerated. Mock inoculated (water only) treatments were included as a control. This experiment was repeated 3 times, with 3 independent replicates for each experiment (*n* = 9). Statistical analysis was evaluated using a two tailed unpaired t test (**** *p* value < 0.00001). **b** CPK3 is not modified by flg22. *cpk3-2*/35S::*CPK3* plants were inoculated with 1 μM flg22, by infiltration. As equal amount of total protein was separated on a 10% Mn^2+^/Phos-tag^TM^ gel. **c**, **d** Immunocomplex assay. For kinase assays, myc-CPK3 was immuno-precipitated with an anti-myc antibody after inoculation of flg22 (c) and *Pst* DC3000 (d) in *cpk3-2*/35S::*CPK3* and incubated with MBP in kinase buffer. **b-d**, Experiments were repeated 3 times.

**Supplementary Figure 9.** Quantitative real-time PCR analysis in WT Col-0, the *cpk3-2* mutant, and *CPK3* complementation lines. Analysis of defense and immune-related mRNA expression in Arabidopsis lines following flg22, *Pst* DC3000, *Pst* DC3000-AvrPphB, and *Pst* DC3000-AvrPphB^C98S^ infiltration. Transcript levels of PTI-associated transcripts were quantified by qPCR, **a** *FRK1*, **b** *PR-1*, **c** *WRKY70*, and **d** *WRKY46*. mRNA levels were normalized against the endogenous expression of protein phosphatase 2A (*PP2A)*. Values shown are the average of three independent biological replicates (*n* = 3), and error bars represent SEM. Letters indicate statistically significant difference, as determined by a one-way ANOVA (*p* value < 0.05; ns, not significant).

**Supplementary Figure 10.** Loss of *CPK3* does not alter the cellular ratio of globular (G)- and filamentous (F)-actin in response to **a** *Pst* DC3000 infection and **b** flg22 elicitation. Filamentous (F)-actin and globular (G)-actin in plant cells was quantified using a G-actin/F-actin in vivo assay kit. **c** Actin loading standards for relative quantification of G- and F-actin populations from elicited Arabidopsis leaves processed in **a** and **b**. Experiments were repeated 2-3 times.

**Supplementary Figure 11.** Flg22-induced stomata closure in the *cpk3-2* mutant complementation lines Stomatal apertures (width/length) were calculated using ImageJ. (Mann-Whitney U-test; **** *p* < 0.0001; ns, not significant). Error bars represent means ± SD from three independent biological repeats (185, 158, 173, and 161 images were collected respectively within total three biological repeats).

**Supplementary Figure 12.** Identification and characterization of *cpk* SALK mutant lines. **a** Identification of individual T-DNA insertion lines from the Arabidopsis Biological Resource Center (ABRC). DNA primers were designed using http://signal.salk.edu/tdnaprimers.2.html. **b-g** Bacterial growth curve analysis with various *Pst* DC3000 strains taken at 0 dpi to ensure equal initial inoculum. Values are means ± SE (one-way ANOVA, *n* = 3). **h-k** Bacterial growth curve analysis at 4 dpi using various *Pst* DC3000 strains. This experiment was repeated 3 times, with 3-5 plants for each experiment. Values are means ± SE (one-way ANOVA, *p* value < 0.05; *n* = 9-15).

**Supplementary Figure 13.** Generation and disease resistance evaluation of multiple *cpk3-2* mutant complementation lines. **a** qRT-PCR analysis of mRNA levels of *CPK3* and confirmation of *cpk3-2* mutant complementation lines. Error bars represent means ± SD from two technical replicates of three independent biological repeats (*n* = 6). **b** Western blot analysis (anti-Myc) of the complemented *cpk3-2* mutant (i.e., *cpk3-2/35S::CPK3*). **c** Bacterial growth curve analysis in the *cpk3-2/35S::CPK3* complementation lines by syringe-infiltration at 3 dpi. Values are means ± SE (one-way ANOVA, *p* value < 0.05; *n* = 9). **d** Bacterial growth curve analysis in the *adf4-2* and *cpk3-2/adf4-2* lines by syringe-infiltration at 3 dpi. Values are means ± SD (one-way ANOVA, *p* value < 0.05; *n* = 13, 7, 12 and 6 from *Pst* DC3000 inoculated samples; *n* = 20, 22, 20 and 17 from *Pst* DC3000-AvrPphB inoculated samples).

**Supplementary Figure 14.** PBS1 nor RPS5 are physically associated with the actin cytoskeleton. Co-immunoprecipitation of **a** HA-PSB1^K115N^ and **b** RPS5-HA with anti-actin antibodies. Co-immunoprecipitation experiments were performed using Agrobacterium transient expression (40 h) in *N. benthamiana*. Total protein extracts containing epitope-tagged proteins (HA-PBS1^K115N^ and RPS5-HA) were immunoprecipitated using monoclonal antibodies to Arabidopsis actin and the resultant pull downs were denatured and separated by SDS-PAGE, followed by western blot analysis. These experiments were repeated three times with similar results.

**Supplementary Figure 15.** *Pst* DC3000-AvrPphB inoculation leads to a disruption in actin cytoskeletal architecture in WT Col-0, preventing quantitative analysis of actin filament organization. **a** Four-week-old Col-0/*35S::GFP-fABD2* plants were dip-inoculated with *Pst* DC3000-AvrPphB (10^7^ CFU mL^-1^) and incubated at room temperature (ca. 22 °C) for 24 h. Infiltrated leaves were analyzed by laser-scanning confocal microscopy. This experiment was repeated twice (*n* = 20 leaves) with similar results. Representative images are shown. **b** *In vitro* analysis of G- and F-actin (non-muscle F-actin) ratios following incubation with recombinant AvrPphB. **c** AvrPphB does not alter the polymerization activity of G-actin. Purified AvrPphB and AvrPphB^C98S^ were incubated with non-muscle F-actin for 30 minutes. The supernatant (S) and pellet (P) fractions were separated by ultracentrifugation. Proteins were separated by SDS-PAGE and stained with Coomassie brilliant blue. b-c, These experiments were repeated 2 times.
